# Demonstration of an enhanced dosing pattern for debulking large and bulky unresectable tumors via differential hole‐size spatially fractionated radiotherapy

**DOI:** 10.1002/acm2.70127

**Published:** 2025-05-27

**Authors:** Joshua Misa, William St. Clair, Damodar Pokhrel

**Affiliations:** ^1^ Department of Radiation Medicine Medical Physics Graduate Program University of Kentucky Lexington Kentucky USA

**Keywords:** hole‐size, hole‐spacing, indirect cell kill, large and bulky tumors, lattice pattern, SFRT

## Abstract

**Purpose/objective:**

We propose a novel lattice deployment for spatially fractionated radiotherapy (SFRT) treatments. In this approach, a larger diameter high‐dose sphere is centrally placed in the bulky tumor mass and surrounded by smaller diameter high‐dose spheres.

**Materials/methods:**

Thirty SFRT patients (10 head and neck [HN], 10 abdominal/pelvis, and 10 chest/lung cases) treated with an MLC‐based crossfire method were retrospectively analyzed. Eleven differential hole‐size lattice patterns were benchmarked against the clinically delivered SFRT plans (1 cm diameter cylinders, 2 cm spacing) and the standard uniform lattice pattern (1.5 cm diameter spheres, 3 cm spacing). These patterns varied in core diameter (C: 2–4 cm), spacing (S: 2–4 cm), and peripheral diameter (P: 1–2 cm). In addition to peak‐to‐valley‐dose ratio (PVDR), tumor dose metrics (D_50%_, V_50%_, D_mean_)_,_ D_max_ to nearby critical organs, and ablative dose (V_75%_/V_50%_ and V_15Gy_) were evaluated.

**Results:**

10 out of 11 differential hole‐size patterns showed increases in D_50%_, D_mean_, and V_50%_ compared to the standard lattice pattern. One pattern (C = 3 cm, S = 2 cm, P = 1.5 cm) outperformed the clinical SFRT plans in D_50%_ (Δ = 1.8 Gy, *p* = 0.003; Δ = 2.0 Gy, *p* = 0.015; Δ = 0.9 Gy, *p* = 0.045), D_mean_ (Δ = 1.6 Gy, *p* = 0.003; Δ = 1.7 Gy, *p* = 0.021; Δ = 0.7 Gy, *p* = 0.042), and V_50%_ (Δ = 20.4%, *p* < 0.001; Δ = 16.6%, *p* = 0.008; Δ = 10.3%, *p* = 0.079) for the HN, abdominal/pelvis, and chest/lung SFRT patients, respectively. This pattern also demonstrated average increases to D_5%_ D_10%_, D_90%_ across all 30 patients compared to both benchmarked patterns. However, this pattern showed reduced PVDR compared to the clinical and standard SFRT plans but still achieved a ratio > 3.0. All differential hole‐size patterns demonstrated decreases in D_max_ to critical organs compared to the clinical SFRT plans. Moreover, compared to the clinical SFRT and the standard lattice plans, 9 out of 11 differential hole‐size patterns demonstrated increases in V_75%_/V_50%_ and V_15Gy_.

**Conclusion:**

All differential hole‐size SFRT replans were clinically acceptable, with C = 3 cm, S = 2 cm, and P = 1.5 cm providing the optimal setting for select tumors. Differential lattice patterns enhanced the ablative dose to the bulky tumors while restricting the maximum dose to adjacent critical organs.

## 1 INTRODUCTION

Spatially fractionated radiotherapy (SFRT), also commonly known as GRID therapy, is a technique that breaks the standard convention of radiation therapy treatments for large and bulky unresectable tumors. Typically, tumors are irradiated with a uniform dose distribution to ensure all tumor cells receive adequate doses. However, SFRT utilizes a highly heterogeneous dose distribution, in which the tumor receives an alternating pattern of high and low‐dose regions. The utility of this technique is seen for large, bulky, and unresectable tumors typically greater than 6 cm in diameter. It is advantageous for these cases as it can deliver an ablative dose to the tumor while respecting nearby critical organs’ dose limits, especially the skin.^1^, ^2^ SFRT has had favorable clinical outcomes for a variety of treatment sites. A study from Huhn et al.^3^ investigating bulky head and neck cancer treatment by combining a curative course of radiation therapy with GRID therapy observed a local tumor control rate of 92%. Another study from Mohiuddin et al.^4^ reported a 91% overall response rate for a wide range of treatment sites (pelvis, abdomen, extremities, thorax, and head and neck) in the palliative setting while maintaining normal tissue toxicity. In addition to the ablative direct cell kill that SFRT can deliver, regions of low doses are hypothesized to benefit from indirect cell death. The three main hypothesized indirect cell‐death mechanisms attributed to the utility of SFRT are radiation‐induced bystander effect, intratumor immune response, and tumor microvasculature damage.^5^, ^6^, ^7^, ^8^, ^9^, ^10^ Traditionally, SFRT has been delivered with a single‐field physical GRID block slotted onto the LINAC head.^11^, ^12^ Many downsides are attributed to the traditional physical GRID block, such as its difficulty in sparing adjacent critical organs and its heavy weight, which poses a concern for both therapists and patients. It is also not commonly implemented into the treatment planning system (TPS), making it difficult for physicians and planners to evaluate SFRT plans. The continued rise in SFRT has shifted away from the physical GRID block with the development of new clinical implementations such as MLC‐based method, VMAT, Helical Tomotherapy, and proton therapy.^13^, ^14^, ^15^, ^16^, ^17^, ^18^, ^19^, ^20^

**FIGURE 1:**
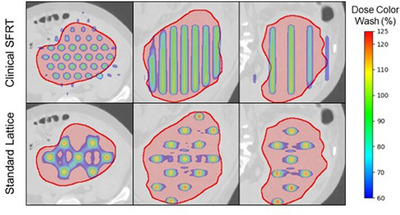
Demonstration of the highly heterogeneous dose distributions via clinical SFRT pattern (top panels) and uniform hole‐size and spacing lattice pattern (bottom panel). The axial (left), coronal (middle), and sagittal (right) views of a previously clinically delivered SFRT plan for a large pelvis mass (red contour) with a diameter of 16 cm are shown. The 60%–125% isodose color wash is displayed relative to the nominal prescription dose of 15 Gy, comprised within the target in the lattice SFRT plan.

The two commonly used patterns for SFRT are the GRID pattern and the Lattice pattern. The GRID patterns are characterized by the cylindrical dose distribution delivered to the tumor, while the lattice pattern is characterized by its spherical dose distribution, as shown in Figure 1. Similar to traditional single‐field block, the GRID patterns typically deliver dose distributions with uniform 1–2 cm diameter high‐dose cylinders with a 2–3 cm center‐to‐center spacing.^21^, ^22^ Lattice patterns generally are constructed in a hexagonal structure, with a sphere diameter ranging from 1–2 cm and the distances between nearby spheres range from 2–5 cm.^22^, ^23^ Our clinic utilizes an SFRT planning method called the cross‐fire method, a forward‐planning technique that uses MLC leaves fitting algorithm to generate a GRID pattern.^13^, ^24^, ^25^ While keeping consistent with the traditional approach, the MLCs are fitted with a pattern of 1 cm openings and 1 cm closings, creating 1 cm diameter high‐dose rods with center‐to‐center spacing of 2 cm at isocenter. Due to being a forward planning method, the MLC crossfire technique allows SFRT treatments to be delivered on the same day as CT simulation. However, utilizing an inversely planned lattice pattern via VMAT allows for superior normal tissue sparing and more flexibility in generating heterogeneous tumor dose distribution.^14^, ^26^ The challenge of lattice therapy is that it is a complex and resource‐intensive planning process that includes lattice contouring, inverse plan optimization, and patient‐specific VMAT quality assurance that may not allow for same‐day SFRT treatment.

To date and to the best of our knowledge, SFRT patterns have utilized a constant uniform diameter hole‐size and hole‐spacing throughout the bulky tumor when contouring. This study aims to investigate a novel SFRT pattern deployment that doesn't use a constant diameter lattice sphere contour but a differential lattice sphere diameter for contouring inside the tumor mass. We propose a series of patterns where the center of the tumor has a large‐diameter high‐dose sphere, surrounded by smaller‐diameter high‐dose spheres towards the periphery of the tumor. We hypothesize that this pattern will give a higher ablative dose to the core of the tumor while being able to maintain the dose outside the tumor—potentially sparing adjacent critical organs. This is important as it may allow for escalating doses to be delivered to the center of the tumor that typically may be more hypoxic and/or radioresistant. Our differential hole‐size method is analogous in achieving the same goal as SBRT‐PATHY and SCART, where a large ablative dose is to be delivered to the central part of the tumor while maintaining low dose levels towards the periphery.^27^, ^28^ In contrast, the novelty of our methodology is that we retain the alternating pattern of high‐ and low‐dose characteristic of SFRT dose distribution with the differential lattice configuration to still deliver the ablative doses towards the periphery of the tumor while still restricting the maximum dose to adjacent critical organs. This non‐uniform pattern may be advantageous for cancer types that are more radioresistant, such as sarcomas or renal cell carcinomas, as it allows for a higher ablative dose to be delivered, encouraging further tumor debulking and sensitization for follow‐up combination therapy post‐SFRT. This SFRT lattice pattern generation will introduce another method that planners and attending physicians could use to enhance their patients’ care via escalating SFRT dose to large and bulky tumors.

## 2 MATERIALS AND METHODS

### 2.1 Patient cohort and treatment planning

Under the institutional review board‐approved protocol, 30 patients previously treated with SFRT, followed by conventional radiation therapy, were included in our retrospective simulation study cohort. This cohort consisted of 10 head and neck (HN) patients, 10 abdominal/pelvis patients, and 10 lung/chest patients with large tumor masses. The entirety of the cohort was set up in the head‐first‐supine position, and 3D axial planning CT images were acquired with our clinic's site‐specific patient setup and immobilization protocols on SOMATOM go.Open PRO CT scanner (Siemens Healthineers, Malvern, PA). The attending physician(s) drew the SFRT gross tumor volume (GTV) on the 3D planning CT image set in the Eclipse TPS, with nearby critical organs also delineated. Patients were selected based on whether they had been previously treated with SFRT and if they had a tumor diameter greater than 6 cm. The HN patients had an average tumor diameter of 10.17 cm (6.5–13.3 cm) and a corresponding tumor volume of 548.3 cc (142.4–1235.5 cc), and the abdominal/pelvis patients had an average tumor diameter of 10.7 cm (8.7–16.0 cm), thus relatively larger tumor volume of 719.3 cc (347.79–2150.58 cc). Similarly, the chest and lung patients had an average tumor diameter of 8.0 cm (6.0–12.4 cm) with corresponding average tumor sizes of 318.8 cc (115.7–1007.0 cc). All thirty patients had their clinical SFRT treatments planned using the 3D‐conformal MLC‐based Crossfire technique^13^, ^24^, ^25^ and were prescribed for a nominal single dose of 15 Gy to GTV with no additional margin. As mentioned, this is a forward‐planning technique that our clinic adopted to allow SFRT treatments to be delivered on the same day as CT simulation for immediate patient care. These plans used MLCs fitted to the GTV in an alternating pattern to generate 1 cm diameter high‐dose rods with 2 cm center‐to‐center spacing in the plane of isocenter. This technique utilizes up to 6 coplanar crossfire gantry angles 60° apart with a 90° collimator setting with the option to use differentially weighted 6 or 10 MV flattened beams. Additionally, wedged fields and field‐in‐field approaches are sometimes used to improve normal tissue sparing for these clinical cases.

### 2.2 Lattice deployment method

Utilizing the clinical dataset of anonymized planning CT images with SFRT GTV contours and nearby OAR structures, the lattice deployment follows the guidelines described by Duriseti et al.^14^ However, with the slight adjustment in that we placed the initial lattice vertex in the centroid of the GTV. Based on the SFRT planning guidelines, a hexagonal lattice pattern was populated around this initial vertex. If performed manually, this would be achieved by overlaying a grid guide onto the GTV, and the remaining vertices are to be contoured using a 3D brush and placed onto the grid intersections. Our clinic has developed an Eclipse API script that automatically places these vertices within the GTV based on this SFRT planning guideline. An example of this lattice deployment technique is shown in Figure 1.

### 2.3 Differential‐hole size plans

**FIGURE 2:**
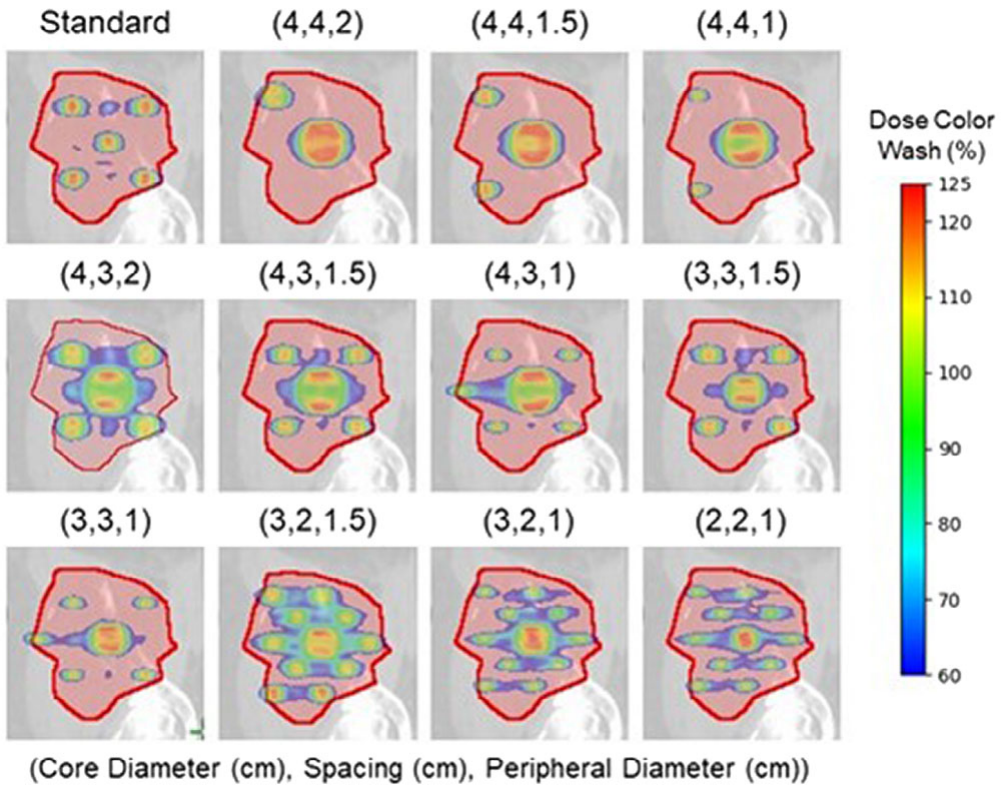
Example of deploying the 12 different SFRT lattice patterns and the resulting isodose distributions (60%–125% isodose color wash of 15 Gy contained within the tumor) investigated in this study. The Core diameter (cm), Spacing (cm), and Peripheral diameter (cm) are shown in parenthesis above each lattice pattern to indicate the differential lattice deployment parameters for a right pelvis mass. The standard lattice pattern utilizing a constant vertex diameter of 1.5 cm, and a spacing of 3 cm is shown.

Eleven differential hole‐size patterns were automatically generated and investigated in this simulation study, and one standard lattice pattern was used to assist in benchmarking. These 11 differential lattice patterns consisted of 3 different high dose core diameters (C) of 4 cm, 3 cm, and 2 cm, peripheral high dose sphere diameters (P) ranging from 1 to 2 cm, and spacings (S) ranging from 2 to 4 cm. Spacing here refers to the grid guide dimensions used when deploying the lattice spheres as mentioned above. The standard lattice pattern consisted of all 1.5 cm diameter spheres with S of 3 cm. The deployment of the lattice contours utilized for the differential‐hole size SFRT plans follows the methodology outlined above. The key difference is that the initial lattice vertex would be a sphere of diameter C, while all subsequent lattice vertices would be a sphere of diameter P. Figure 2 demonstrates all 12 patterns and how they are deployed inside a tumor volume. All 12 patterns were attempted to be replanned on all 30 patients in the cohort. These SFRT patterns were replanned with Varian's Eclipse TPS (v.16.12) and calculated with the AcurosXB (v.16.12) dose engine (Varian Medical Systems, Palo Alto, CA). All original clinical SFRT plans and lattice replans were generated to be delivered on the Varian TrueBeam LINAC equipped with Millennium 120MLC. All replans consisted of four full VMAT arcs with a beam energy of 6MV‐FFF and collimator angles at ± 15°. Across all 12 replans for each patient, the same optimization objectives and priorities were utilized. Lastly, after the final dose calculation, all replans were re‐normalized, so the maximum dose to the target was 125% of the prescription dose, 15 Gy–similar to our SBRT planning clinical guidelines. Following the treatment planning approach outlined by Grams et al.,^26^ a VMAT optimization was implemented such that 50% of the lattice spheres received the prescription dose of 15 Gy, which remained true even after plan re‐normalization. A replan was performed for all lattice therapy plans if more than one sphere could be placed within the GTV target.

### 2.4 Data collection & analysis

The eleven differential hole‐size patterns were compared against the standard lattice pattern and benchmarked against the clinically delivered cylindrical SFRT pattern. The tumor dose metrics selected to be analyzed were D_50%_, D_mean_, and V_50%_ extracted from the SFRT GTV target. Additionally, as recommended by the Radiosurgical Society (RSS) working group on SFRT, we extracted GTV D_5%_, D_10%_, D_90%_, and PVDR (D_10%_ ÷ D_90%_).^29^ Selected immediately adjacent organs‐at‐risk (OARs) were evaluated based on the treatment site. For instance, the maximum dose to the spinal cord, esophagus, and larynx was evaluated for HN patients. For abdominal/pelvis SFRT patients, the maximum dose to the bowel, bladder, and rectum was assessed. Lastly, for chest/lung patients, the maximum dose to the spinal cord, esophagus, and heart was evaluated. Additionally, D_2cm_, the maximum dose at 2 cm away in any direction from the SFRT tumor which indicates the dose fall off from the tumor boundary, was analyzed for all cases. Two new metrics were utilized to evaluate the ablative doses to the gross tumor. Due to the lattice pattern of SFRT, only a small percentage of the GTV receives a high dose. We devised a new metric to analyze how much of these high‐dose regions receive the ablative doses. The V_75%_/V_50%_ × 100 will indicate how much of the high‐dose volume the tumor receives (V_50%_) is associated with ablative doses (V_75%_), defined as ablative volume percentage (AVP). Here, we utilized 75% and 50% of the prescription to define AVP, as SFRT plans are typically prescribed to 15–20 Gy in 1 fraction; thus, 75% of the prescription dose is within the ablative dose range. However, the AVP metric might not be applicable for treatment plans prescribed at a lower dose level, as it will not indicate an ablative dose and may not qualify as an SFRT plan. The other new metric we analyzed is V_15Gy,_ which provides information on how much of the tumor is receiving the peak region of the SFRT dose distribution. A student‐paired *t*‐test was performed against the clinical and standard lattice pattern SFRT plans for all differential hole‐size patterns. A statistical significance level was defined at *p‐*value < 0.05.

## 3 RESULTS

### 3.1 Sphere packing

**FIGURE 3:**
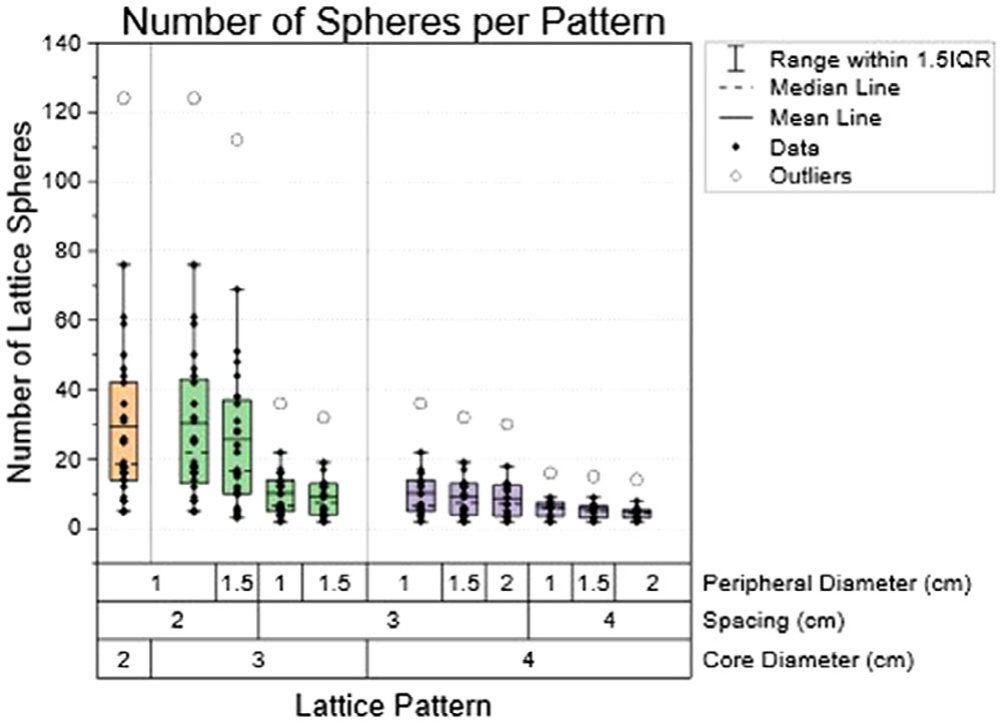
Boxplot representing how many lattice spheres can be packed for each pattern across the SFRT patient cohort simulated in this study. The color of the boxes represents the different core diameters (in cm) for the differential lattice patterns. The box plot displays the mean line, median line, individual data points, outliers, and the range within 1.5, the interquartile range on either side of each pattern's first and third quartile.

**FIGURE 4:**
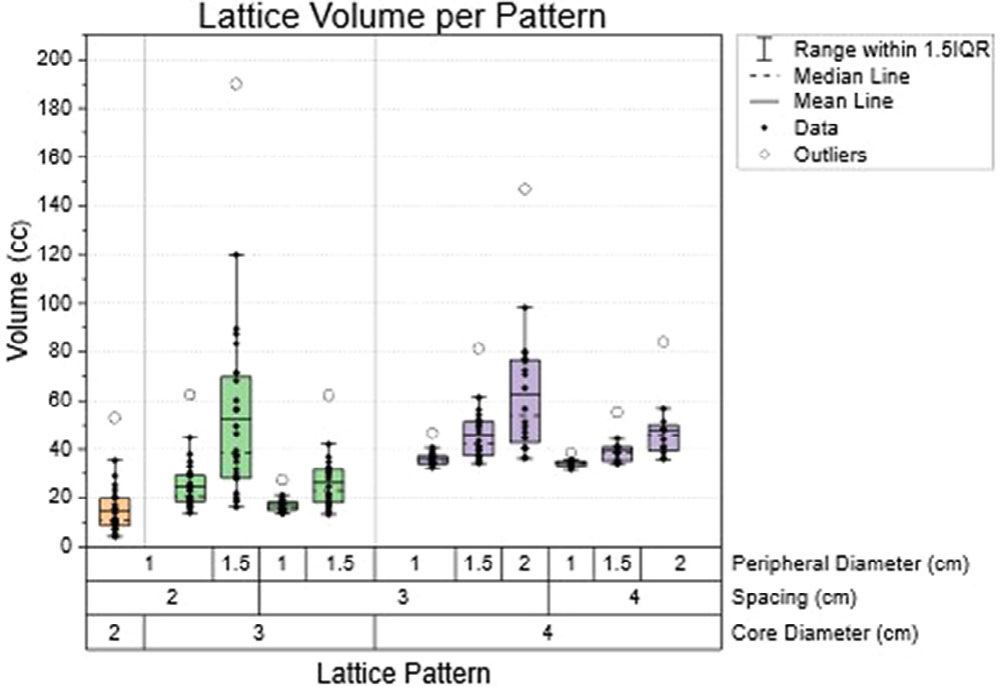
Boxplot representing the total lattice volume each pattern was able to produce for the entire SFRT patient cohort. The color of the boxes represents the different core diameters (in cm) for the lattice patterns. The box plot displays the mean line, median line, individual data points, outliers, and the range within 1.5, the interquartile range on either side of each pattern's first and third quartile.

**FIGURE 5:**
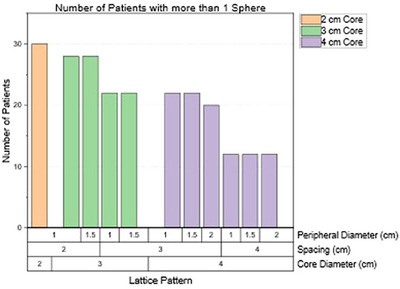
A bar plot showing how many patients could be replanned with more than one sphere packed within the tumor for each pattern in this cohort. The color of the boxes represents the different core diameters (in cm) for the lattice patterns.

Figure 3 shows the total number of lattice spheres packed for each pattern for all 30 patients. This total accounts for both the core sphere and all peripheral spheres. As expected, more spheres can be packed in the SFRT target size with smaller spacing and peripheral diameters. However, the total number of spheres isn't adequate to quantify the lattice deployment. Figure 4 demonstrates the total lattice volume within the target for each pattern. Even though the patterns with 2 cm spacing resulted in the highest number of spheres, they did not provide the largest lattice volume within the tumor volume. On average, the two patterns resulting in the largest lattice volume were C = 4 cm, S = 3 cm, P = 2 cm, and when C = 3 cm, S = 2 cm, P = 1.5 cm. In this study, we only considered generating SFRT plans that could pack more than one sphere within the target. The data analysis didn't include cases where the pattern couldn't pack more than one sphere. As shown in Figure 5, we observe that the smaller the core diameter, the more likely it is that more than one sphere can be packed. This is because, for some smaller and elongated tumors, the centroid of the tumor could only tolerate a 2 cm core diameter sphere. Additionally, the smaller the spacing between the spheres utilized, the more likely an SFRT target can have more than one sphere packed when using these differential lattice patterns.

### 3.2 Tumor metrics

**FIGURE 6:**
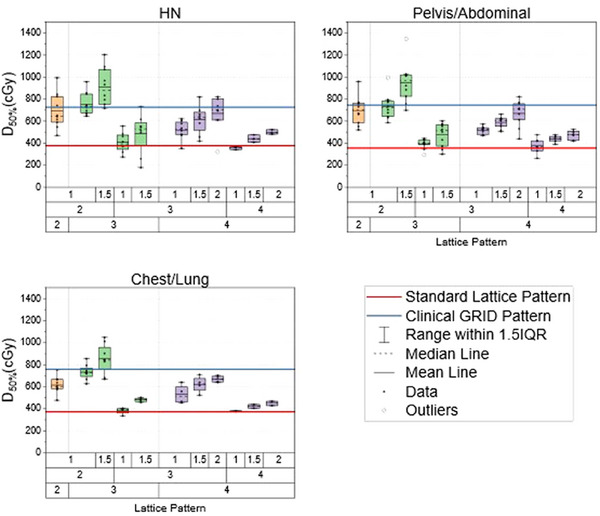
Three box plots demonstrating each lattice pattern's D_50%_ for the HN site (top left), pelvis (top right), and chest/lung site (bottom left) used in this SFRT cohort. The color of the boxes represents the different core diameters of each pattern used. Orange color represents a 2 cm core diameter, green represents a 3 cm core diameter, and purple represents a 4 cm core diameter.

Analyzing D_50%_, one lattice pattern showed both a clinical and statistical improvement when compared against the clinically delivered cylindrical SFRT pattern across all three treatment sites. The pattern of C = 3 cm, S = 2 cm, and P = 1.5 cm showed statistically significant increase in D_50%_ for HN tumors (9.09 ± 1.72 Gy vs. 7.26 ± 0.75 Gy, *p‐*value = 0.003), abdominal/pelvis masses (9.51 ± 1.82 Gy vs. 7.46 ± 0.54 Gy, *p‐*value = 0.015), and chest/lung sites (8.54 ± 1.37 Gy vs. 7.60 ± 0.75 Gy, *p‐*value = 0.045). Figure 6 shows how all the differential hole‐size patterns compare against the clinical SFRT and standard uniform lattice patterns. The trend shown is an increase in D_50%_ with decreasing spacing between the spheres. Additionally, with increasing peripheral or core dose diameter, there is a resulting increase in D_50%_.

**FIGURE 7:**
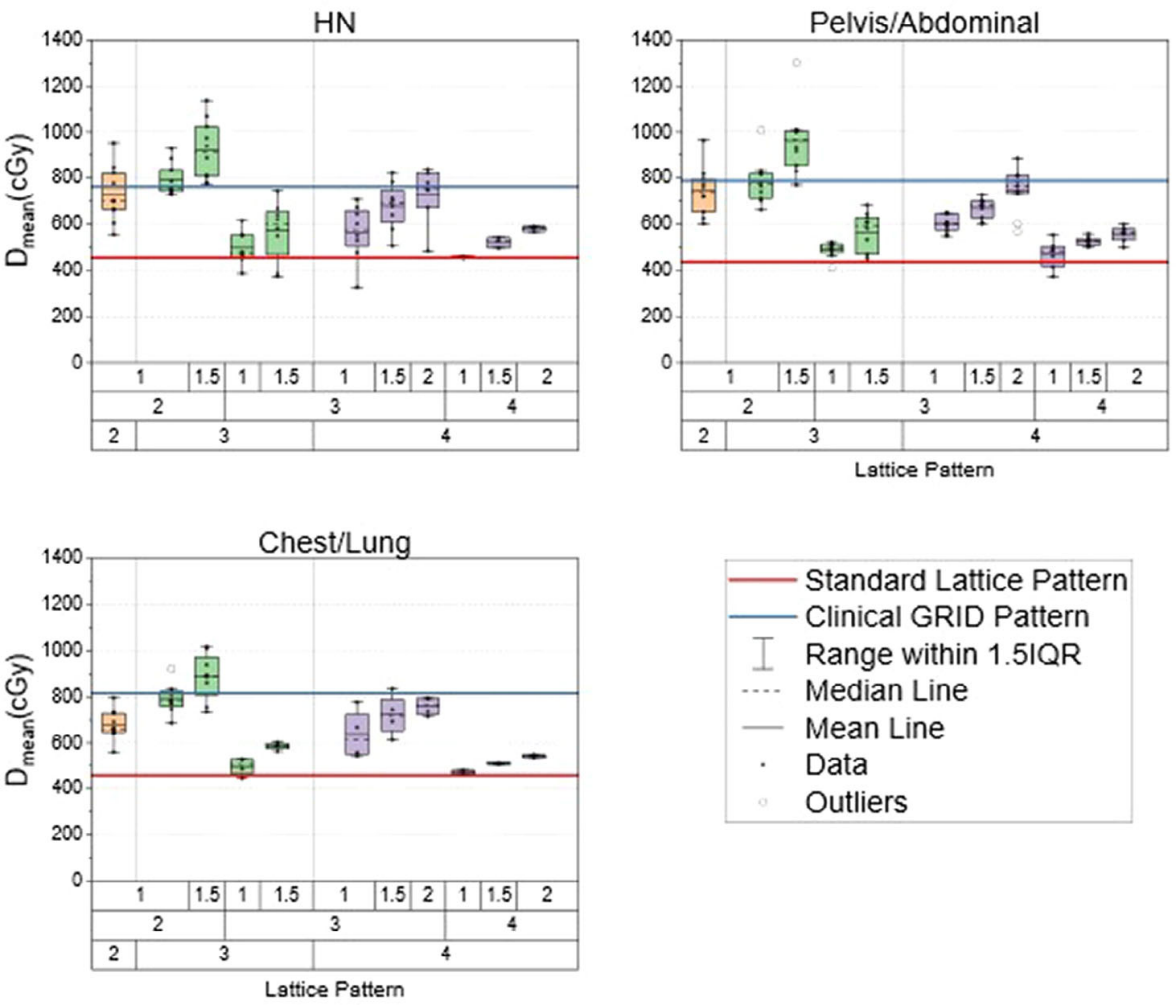
Three box plots demonstrating each pattern's D_mean_ for the HN site (top left), Pelvis (top right), and chest/lung site (bottom left) used in this SFRT cohort. The color of the boxes represents the different core diameters of each pattern used. Orange color represents a 2 cm core diameter, green represents a 3 cm core diameter, and purple represents a 4 cm core diameter.

The same differential lattice pattern as before (C = 3 cm, S = 2 cm, P = 1.5 cm) remains the only pattern that shows a statistical improvement to D_mean_ when compared against the clinically delivered SFRT pattern. The improvement is demonstrated for HN patients (9.21 ± 1.28 Gy vs. 7.63 ± 0.79 Gy, *p‐*value = 0.003), pelvis patients (9.63 ± 1.45 vs. 7.89 ± 0.71 Gy, *p‐*value = 0.021), and chest/lung patients (8.85 ± 1.05 Gy vs. 8.15 ± 0.79 Gy, *p‐*value = 0.042). Figure 7 depicts these results alongside all the other patterns benchmarked against the clinically delivered SFRT treatments. Similar trends were observed when analyzing D_50%_ and D_mean_.

**FIGURE 8:**
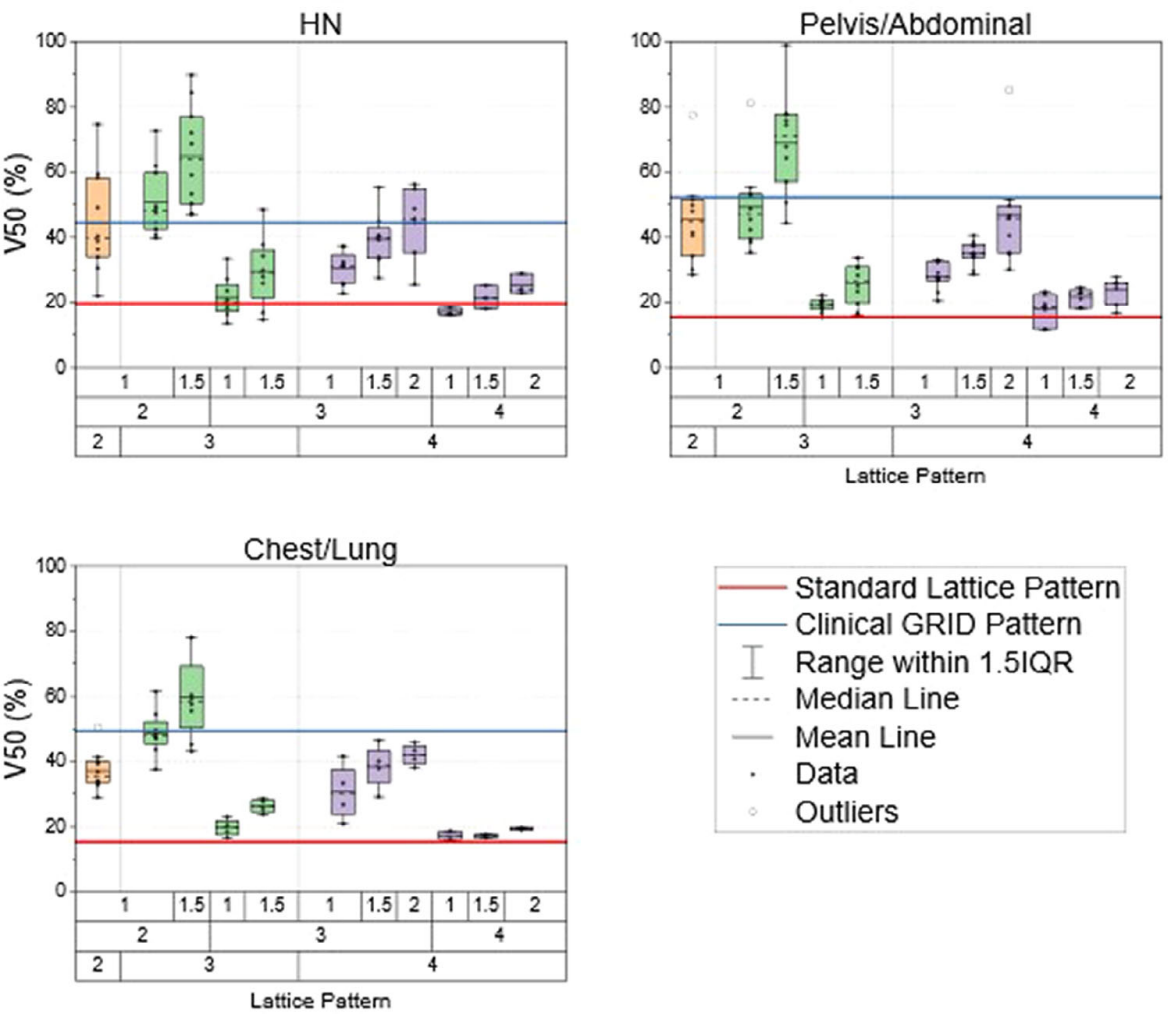
Three box plots demonstrating each pattern's V_50%_ for the HN site (top left), pelvis (top right), and chest/lung site (bottom left) in this SFRT patient cohort. The color of the boxes represents the different core diameters of each pattern used. Orange represents a 2 cm core diameter, green represents a 3 cm core diameter, and purple represents a 4 cm core diameter.

Again, one lattice pattern (C = 3 cm, S = 2 cm, P = 1.5 cm) statistically increased V_50%_ for HN and abdominal/pelvis cases. In comparison against the clinically delivered SFRT pattern, this pattern saw a statistical rise in V_50%_ for HN patients (64.9 ± 15.7% vs. 44.5 ± 5.8%, *p‐*value < 0.001) and pelvis tumors (68.9 ± 15.8% vs. 52.3 ± 5.3%, *p‐*value = 0.008). Though not statistically significant, this lattice pattern, on average, increased V_50%_ for chest/lung cases (59.7 ± 13.0% vs. 49.4 ± 6.8%, *p‐*value = 0.079). Figure 8 demonstrates how the differential hole‐size patterns are compared against the clinical SFRT treatments and the standard lattice pattern. Like what was found before with the other two dose metrics, the same trends were observed when analyzing V_50%_.

In addition to the clinically delivered SFRT plans, the 11 patterns were benchmarked against the standard lattice pattern. This is represented by the red line in Figures 6, 7, and 8. Across all treatment sites, all lattice patterns except one demonstrated an average increase in D_mean_, D_50%_, and V_50%_. Notably, the one lattice pattern (C = 4 cm, S = 4 cm, P = 1 cm) has shown an average decrease in D_50%_ and V_50%_ compared to the standard lattice pattern for HN cases. Six of the 11 differential‐hole size patterns showed a statistical increase in these metrics across all three treatment sites compared to the standard lattice pattern. These six patterns were when C = 4 cm and S = 3 cm or 2 cm, or when C = 3 cm, S = 3 cm, and P = 1.5 cm.

Table 1 reports the extracted GTV dose metrics for all the differential hole‐size plans, clinical SFRT plans, and standard lattice plans across the patient cohort. Similar to the trends shown with D_50%_, D_mean_, and V_50%_, the recommended dose metrics of D_5%_, D_10%_, and D_90%_ increase with increasing peripheral or core diameter size and decreasing spacing.

**TABLE 1:** Extracted GTV target dose metrics averaged across all three investigated SFRT treatment sites, specific dosimetric parameters were selected as recommended by the RSS SFRT white paper.^29^ The PVDR is defined as D_10%_÷D_90%_. Reported is the average ± standard deviation.

Lattice Pattern				Dose Metric				
Core diameter (cm)	Spacing (cm)	Peripheral diameter (cm)	D_5%_ (Gy)	D_10%_ (Gy)	D_50%_ (Gy)	D_mean_ (Gy)	D_90%_ (Gy)	PVDR
4	4	2	14.90 ± 0.88	11.23 ± 0.98	4.76 ± 0.36	5.64 ± 0.30	1.53 ± 0.65	10.65 ± 9.92
		1.5	14.30 ± 1.30	10.72 ± 1.01	4.36 ± 0.30	5.21 ± 0.20	1.22 ± 0.49	12.06 ± 10.18
		1	13.62 ± 2.08	10.54 ± 2.32	4.14 ± 1.14	4.69 ± 0.36	1.17 ± 0.75	11.30 ± 5.30
	3	2	15.50 ± 1.00	13.75 ± 1.55	6.65 ± 1.09	7.47 ± 1.02	2.33 ± 1.23	9.54 ± 9.19
		1.5	15.18 ± 0.99	13.20 ± 1.53	6.01 ± 0.81	6.81 ± 0.76	1.98 ± 0.90	9.04 ± 7.37
		1	14.83 ± 1.33	12.59 ± 1.89	5.28 ± 0.54	6.04 ± 0.67	1.50 ± 0.62	10.58 ± 7.15
3		1.5	14.17 ± 0.89	11.41 ± 0.72	5.17 ± 0.87	5.75 ± 0.98	1.76 ± 0.77	9.16 ± 8.28
		1	13.00 ± 1.41	10.28 ± 1.11	4.22 ± 1.09	4.93 ± 0.50	1.39 ± 0.75	10.14 ± 7.76
	2	1.5	15.64 ± 0.73	14.34 ± 0.82	8.99 ± 1.76	9.42 ± 1.45	4.74 ± 1.94	3.83 ± 2.40
		1	14.81 ± 1.02	13.06 ± 0.97	7.40 ± 0.96	7.97 ± 0.87	3.54 ± 1.19	4.32 ± 2.32
2		1	13.63 ± 0.52	11.87 ± 0.59	6.68 ± 1.27	7.25 ± 1.11	3.16 ± 1.19	4.50 ± 2.60
Clinical SFRT plans			13.37 ± 1.21	12.09 ± 1.10	7.44 ± 0.67	7.80 ± 0.79	4.59 ± 1.04	2.91 ± 1.58
Standard lattice plans			11.60 ± 0.92	9.27 ± 1.28	4.22 ± 1.07	4.58 ± 1.40	1.61 ± 0.67	7.17 ± 5.30

### 3.3 Maximum dose to nearby OARs

The three adjacent OARs evaluated for the HN SFRT patients were the spinal cord, esophagus, and larynx. Table 2 shows the resulting D_max_ from all the differential hole‐size patterns, standard lattice patterns, and the clinically delivered MLC‐based SFRT plans. Four lattice patterns saw a statistically significant decrease in maximum dose to all three OARs compared to clinical plans. These patterns occur when a 3 cm spacing was used, and the peripheral diameter was 1 cm or 1.5 cm. However, against the standard lattice pattern, none of the patterns statistically outperformed. All the differential hole‐size patterns saw an average increase in D_max_ compared to the standard lattice pattern of the three OARs. When evaluating D_2cm_, all patterns gave a statistically significant decrease in OAR doses compared to clinical SFRT plans. However, all differential lattice patterns provided an average increase in D_2cm_ compared to standard lattice patterns.

**TABLE 2:** The reported maximum dose to the spinal cord, esophagus, and larynx when utilizing either a differential hole size pattern, clinical SFRT plan, and standard lattice pattern in the HN SFRT cohort. Also included is the maximum dose 2 cm away from the GTV. Reported data are mean ± standard deviation (*p*‐value). Bolded *p*‐values represent statistical significance of *p*‐value < 0.05.

Lattice pattern			D_max_ (Gy)			
Core diameter (cm)	Spacing (cm)	Peripheral diameter (cm)	Spinal cord	Esophagus	Larynx	D_2cm_ (Gy)
4	4	2	1.99 ± 0.24 (0.220)	1.69 ± 1.17 (0.126)	2.58 ± 0.52 (0.079)	5.65 ± 0.07 (**0.012**)
		1.5	2.14 ± 0.25 (0.280)	1.59 ± 1.15 (0.119)	2.60 ± 0.56 (0.075)	5.30 ± 0.38 (**0.004**)
		1	2.07 ± 0.20 (0.276)	1.69 ± 1.25 (0.118)	2.40 ± 0.52 (0.056)	5.50 ± 0.59 (**0.025**)
	3	2	2.52 ± 0.26 (0.072)	2.25 ± 1.28 (0.105)	2.56 ± 1.08 (**0.002**)	5.19 ± 0.60 (**<0.001**)
		1.5	2.43 ± 0.46 (**0.027**)	2.22 ± 1.15 (**0.047**)	2.73 ± 1.15 (**0.002**)	5.23 ± 0.81 (**<0.001**)
		1	2.28 ± 0.36 (**0.011**)	2.01 ± 1.06 (**0.025**)	2.82 ± 1.26 (**<0.001**)	5.18 ± 0.83 (**<0.001**)
3		1.5	2.24 ± 0.36 (**0.009**)	2.13 ± 1.07 (**0.034**)	2.55 ± 1.13 (**0.002**)	5.14 ± 0.80 (**<0.001**)
		1	2.15 ± 0.34 (**0.005**)	2.17 ± 0.74 (**0.044**)	2.88 ± 0.72 (**0.003**)	4.88 ± 0.95 (**<0.001**)
	2	1.5	2.67 ± 0.71 (0.436)	2.96 ± 1.55 (0.213)	3.34 ± 1.71 (**0.034**)	6.03 ± 1.37 (**<0.001**)
		1	2.55 ± 0.67 (0.212)	2.64 ± 1.30 (0.106)	3.15 ± 1.65 (**0.031**)	5.58 ± 0.89 (**<0.001**)
2		1	2.42 ± 0.72 (0.129)	2.55 ± 1.20 (0.090)	3.14 ± 1.59 (**0.031**)	5.61 ± 1.19 (**0.001**)
Clinical SFRT plans			2.92 ± 1.05	3.86 ± 2.21	4.73 ± 2.40	10.66 ± 2.01
Standard lattice plans			2.02 ± 0.40	2.04 ± 1.00	2.33 ± 1.03	4.71 ± 0.88

When evaluating pelvis SFRT plans, the nearby critical organs evaluated were the bowel, bladder, and rectum. Similar to the HN patients’ results, the same four differential hole‐size lattice patterns (S = 3 cm, P = 1.5 cm, or 1 cm) demonstrated a statistically significant decrease in D_max_ for all three OARs mentioned above. Compared to the standard lattice pattern, some patterns did demonstrate decreases in D_max_ to certain OARs, as shown in Table 3. Still, none of the patterns showed statistically significant D_max_ reductions to these three OARs. All differential hole‐size patterns demonstrated statistically significant decreases in D_2cm_ to the clinical SFRT plans, but none statistically outperformed the standard lattice pattern.

**TABLE 3:** The reported maximum dose to the bowel, bladder, and rectum utilizing either a differential hole size pattern, clinical SFRT plans, and standard lattice pattern for pelvis SFRT cohort. Also included is the maximum dose 2 cm away from the GTV. Reported is mean ± standard deviation (*p*‐value). Bolded *p*‐values represent statistical significance of *p*‐value <0.05.

Lattice Pattern			Dmax (Gy)			
Core diameter (cm)	Spacing (cm)	Peripheral diameter (cm)	Bowel	Bladder	Rectum	D_2cm_ (Gy)
4	4	2	4.18 ± 3.04 (**0.006**)	0.68 ± 0.59 (0.158)	1.72 ± 1.01 (0.169)	4.83 ± 1.34 (**<0.001**)
		1.5	4.12 ± 3.00 (**0.005**)	0.62 ± 0.57 (0.126)	1.86 ± 1.19 (0.159)	5.30 ± 0.93 (**<0.001**)
		1	3.72 ± 2.63 (**0.002**)	0.57 ± 0.55 (0.104)	1.51 ± 0.93 (0.177)	5.15 ± 0.99 (**<0.001**)
	3	2	5.52 ± 3.87 (0.098)	1.01 ± 0.72 (0.086)	1.77 ± 1.36 (0.084)	6.12 ± 1.18 (**<0.001**)
		1.5	4.85 ± 3.12 (**0.005**)	1.20 ± 0.77 (**0.043**)	1.82 ± 1.29 (**0.033**)	5.65 ± 0.98 (**<0.001**)
		1	4.15 ± 2.40 **(<0.001**)	1.14 ± 0.81 (**0.035**)	1.74 ± 1.23 (**0.027**)	5.73 ± 0.68 (**<0.001**)
3		1.5	4.70 ± 3.15 (**0.004**)	1.01 ± 0.67 (**0.035**)	1.67 ± 1.19 (**0.028**)	5.42 ± 1.19 (**<0.001**)
		1	3.89 ± 2.36 **(<0.001**)	1.04 ± 0.71 (**0.037**)	1.59 ± 1.15 (**0.023**)	5.26 ± 0.90 (**<0.001**)
	2	1.5	6.06 ± 4.03 (0.333)	2.08 ± 1.10 (0.271)	2.65 ± 1.72 (0.230)	6.76 ± 1.42 (**<0.001**)
		1	5.48 ± 3.64 (0.056)	1.89 ± 1.04 (0.122)	2.25 ± 1.47 (0.102)	6.38 ± 1.46 (**<0.001**)
2		1	5.43 ± 3.52 (**0.028**)	1.71 ± 0.96 (0.099)	2.11 ± 1.39 (0.066)	6.32 ± 1.34 (**<0.001**)
Clinical SFRT plans			6.68 ± 3.31	2.61 ± 1.95	3.58 ± 2.80	11.77 ± 1.36
Standard lattice plans			4.46 ± 2.97	1.16 ± 0.85	1.54 ± 1.12	4.84 ± 0.99

The lung/chest cohort saw no differential hole‐size pattern that statistically outperformed the clinical SFRT plans for all three OARs evaluated (spinal cord, heart, and esophagus). However, on average, all the patterns demonstrated a decrease in maximum dose across these three OARs, as shown in Table 4. Compared to the standard lattice pattern, none of the differential hole‐size patterns showed any statistical improvements to D_max_ to the nearby OARs. On average, all the patterns outperformed the clinically delivered SFRT plans regarding D_2cm_. However, two patterns didn't show statistical significance. This can be attributed to the low sample size for these two patterns for the chest/lung treatment site reported here.

**TABLE 4:** The reported maximum dose to the spinal cord, heart, and esophagus when utilizing either a differential hole size pattern, clinical SFRT plan, and standard lattice patterns for chest/lung SFRT patients. Also included is the maximum dose 2 cm away from the GTV. Reported is mean ± standard deviation (*p*‐value). Bolded *p*‐values represent statistical significance of *p*‐value <0.05.

Lattice Pattern			Dmax (Gy)			
Core diameter (cm)	Spacing (cm)	Peripheral diameter (cm)	Spinal cord	Heart	Esophagus	D_2cm_ (Gy)
4	4	2	1.93 ± 0.80 (0.112)	2.50 ± 2.28 (0.465)	2.46 ± 0.21 (0.132)	4.82 ± 0.59 (0.056)
		1.5	1.79 ± 0.50 (0.169)	2.10 ± 1.90 (0.471)	2.47 ± 0.36 (0.114)	4.09 ± 0.50 (0.056)
		1	2.52 ± 1.51 (0.084)	2.10 ± 1.93 (0.460)	2.57 ± 0.43 (0.108)	5.12 ± 1.33 (**0.015**)
	3	2	2.21 ± 1.18 (0.060)	1.99 ± 2.26 (0.160)	2.47 ± 1.09 (**0.049**)	6.06 ± 1.76 (**0.011**)
		1.5	2.30 ± 1.20 (0.072)	1.78 ± 1.95 (0.171)	2.43 ± 0.98 (0.058)	5.59 ± 1.05 (**0.002**)
		1	2.14 ± 1.05 (0.068)	1.59 ± 1.71 (0.182)	2.31 ± 0.81 (0.057)	5.17 ± 0.78 (**0.003**)
3		1.5	2.23 ± 1.08 (0.067)	1.64 ± 1.81 (0.171)	2.31 ± 0.91 (**0.041**)	5.21 ± 1.12 (**0.002**)
		1	1.95 ± 0.96 (**0.049**)	1.36 ± 1.48 (0.184)	2.20 ± 0.87 (**0.043**)	4.95 ± 0.89 (**0.002**)
	2	1.5	2.60 ± 1.15 (0.661)	2.39 ± 3.06 (0.611)	2.89 ± 0.96 (0.108)	5.91 ± 1.07 (**<0.001**)
		1	2.25 ± 0.99 (0.712)	2.20 ± 2.84 (0.330)	2.75 ± 0.97 (**0.074**)	6.21 ± 1.12 (**<0.001**)
2		1	2.44 ± 0.91 (0.467)	2.18 ± 2.52 (0.094)	2.85 ± 0.88 (**0.078**)	6.02 ± 1.01 (**<0.001**)
Clinical SFRT plans			2.74 ± 1.77	2.94 ± 2.92	3.97 ± 2.23	11.59 ± 2.11
Standard lattice plans			1.86 ± 1.14	1.30 ± 1.63	2.15 ± 0.96	4.85 ± 0.36

Across all three SFRT treatment sites, the 4 cm spacing in most cases produced the lowest D_max_ values for nearby critical organs. The 4 cm spacing differential lattice patterns produced plans with lower D_max_ than the clinically delivered SFRT and standard lattice patterns. However, this wasn't often statistically significant due to the small number of cases that can utilize a 4 cm spacing. Overall, 3 cm spacing produced plans that resulted in more statistically significant reductions to nearby OAR D_max_ compared to clinical SFRT plans. No single pattern outperformed the clinical SFRT plans for all evaluated OARs. Weak trends observed were that D_max_ decreased when the peripheral diameter decreased, core diameter decreased, or when the spacing increased. These trends were weak, as they didn't apply to all sites and/or all OARs evaluated.

### 3.4 Enhanced dosing to the tumor core

To evaluate if these differential lattice patterns enhance the tumor's ablative dose, we analyzed AVP and V_15Gy_. As demonstrated in Tables 5, 6, and 7, all the differential hole‐size patterns provided an average increase in AVP compared to the clinical SFRT plans across all three treatment sites. Compared to the standard lattice pattern, we observed that one pattern, C = 2 cm, on average, had a statistically insignificant decrease in AVP for both abdominal/pelvis and chest sites. Analyzing the V_15Gy_ metric, all differential hole‐size patterns had an average significant increase value compared to clinical SFRT and standard lattice plans, as shown in Tables 5, 6, and 7. As observed in our data analysis, V_15Gy_ and AVP are correlated to the lattice volume size. These two metrics increase with increasing core sphere diameter, decreasing spacing, and increasing peripheral sphere diameter. The two patterns that demonstrated the most significant average increase in these metrics are C = 4 cm, S = 3 cm, P = 2 cm, and C = 3 cm, S = 2 cm, P = 1.5 cm settings. Referring back to Figure 4, these were the top two patterns producing the largest lattice volume.

**TABLE 5:** The resulting AVP (%) and V_15Gy_ (%) of tumor volume treated with differential lattice pattern for HN SFRT cohort. Statistics was reported when compared against both the clinical SFRT plans and standard lattice pattern. Reported is mean ± standard deviation (*p*‐value compared to clinical SFRT, *p*‐value compared to standard lattice pattern). Bolded *p*‐values represent statistical significance of <0.05.

Lattice Pattern			Dose Metric (p‐values)	
Core diameter (cm)	Spacing (cm)	Peripheral diameter (cm)	AVP = V_75%_/V_50%_ × 100 (%)	V_15Gy_ (%)
4	4	2	38.7 ± 4.2 (**0.037**, 0.057)	4.5 ± 0.4 (0.122, **0.003**)
		1.5	36.6 ± 6.8 (0.092, 0.185)	3.6 ± 0.4 (0.205, **0.008**)
		1	37.0 ± 5.0 (0.074, 0.113)	3.0 ± 0.5 (0.372, **0.023**)
	3	2	44.0 ± 4.7 (**0.003**, **<0.001**)	7.3 ± 1.9 (**0.001**, **<0.001**)
		1.5	42.5 ± 6.0 (**0.002**, **<0.001**)	7.0 ± 2.5 (**0.004**, **<0.001**)
		1	40.9 ± 7.4 (**0.011**, **0.001**)	5.9 ± 2.6 (**0.019**, **0.003**)
3		1.5	38.6 ± 6.6 (**0.026**, **<0.001**)	4.5 ± 1.5 (0.070, **<0.001**)
		1	36.2 ± 7.7 (0.083, 0.062)	3.1 ± 1.0 (0.368, **0.010**)
	2	1.5	49.0 ± 8.6 (**<0.001**, **0.025**)	7.1 ± 2.6 (**0.001**, **<0.001**)
		1	37.0 ± 4.2 (**0.012**, 0.436)	4.6 ± 2.5 (**0.049**, **0.010**)
2		1	33.9 ± 5.0 (0.092, 0.942)	2.6 ± 0.8 (0.415, 0.055)
Clinical SFRT plans			28.8 ± 4.7	1.9 ± 2.5
Standard lattice plans			38.7 ± 4.2	1.6 ± 0.5

**TABLE 6:** The pelvis SFRT patient cohort resulting AVP (%) and V_15Gy_ (%). Statistics was reported when compared against both the clinical SFRT plans and standard lattice pattern. Reported is mean ± standard deviation (*p*‐value compared to clinical SFRT plans, *p*‐value compared to standard lattice pattern). Bolded *p*‐values represent statistical significance of <0.05.

Lattice Pattern			Dose Metric (*p*‐values)	
Core Diameter (cm)	Spacing (cm)	Peripheral diameter (cm)	AVP = V_75%_/V_50%_ × 100 (%)	V_15Gy_ (%)
4	4	2	44.5 ± 5.8 (**<0.001**, **0.001**)	5.3 ± 1.6 (**<0.001**, **0.002**)
		1.5	43.0 ± 6.1 (**<0.001**, **0.001**)	4.7 ± 1.8 (**<0.001**, **0.008**)
		1	42.9 ± 5.7 (**<0.001**, **<0.001**)	4.3 ± 1.9 (**0.001**, **0.020**)
	3	2	44.6 ± 6.8 (**<0.001**, **<0.001**)	7.3 ± 1.4 (**<0.001**, **<0.001**)
		1.5	44.2 ± 6.1 (**<0.001**, **<0.001**)	6.2 ± 2.3 (**<0.001**, **<0.001**)
		1	43.4 ± 7.1 (**<0.001**, **0.001**)	5.4 ± 2.4 (**<0.001**, **0.002**)
3		1.5	40.0 ± 6.2 (**<0.001**, **0.003**)	3.8 ± 0.8 (**<0.001**, **<0.001**)
		1	37.2 ± 6.6 (**<0.001**, 0.148)	2.8 ± 1.0 (**<0.001**, **0.019**)
	2	1.5	49.7 ± 11.4 (**<0.001**, **0.023**)	7.4 ± 3.5 (**<0.001**, **<0.001**)
		1	36.3 ± 3.6 (**<0.001**, 0.675)	3.6 ± 0.9 (**<0.001**, **0.010**)
2		1	33.3 ± 3.1 (**0.002**, 0.524)	2.2 ± 0.5 (**<0.001**, **0.038**)
Clinical SFRT plans			26.1 ± 2.8	0.7 ± 0.3
Standard lattice plans			35.1 ± 7.5	1.6 ± 0.5

**TABLE 7:** The chest/lung SFRT patient cohort resulting AVP (%) and V_15Gy_ (%). Statistics was reported when compared against both the clinical SFRT plans and standard lattice pattern. Reported is mean ± standard deviation (*p*‐value compared to clinical SFRT plans, *p*‐value compared to standard lattice pattern). Bolded *p*‐values represent statistical significance of <0.05.

Lattice Pattern			Dose Metric (*p*‐values)	
Core diameter (cm)	Spacing (cm)	Peripheral diameter (cm)	AVP = V_75%_/V_50%_ × 100 (%)	V_15Gy_ (%)
4	4	2	45.4 ± 4.6 (0.139, 0.134)	5.0 ± 0.8 (0.177, 0.118)
		1.5	44.6 ± 4.8 (0.151, 0.148)	4.4 ± 1.0 (0.237, 0.176)
		1	41.8 ± 2.5 (0.104, 0.093)	3.8 ± 1.1 (0.306, 0.252)
	3	2	48.0 ± 5.5 (**0.001**, **0.003**)	9.5 ± 4.4 (0.087, **0.041**)
		1.5	46.9 ± 6.5 (**0.003**, **0.002**)	8.2 ± 4.9 (0.189, 0.081)
		1	46.6 ± 8.8 (**0.014**, **0.008**)	7.7 ± 5.6 (0.256, 0.131)
3		1.5	41.6 ± 7.1 (**0.016**, **<0.001**)	4.5 ± 2.0 (0.613, 0.077)
		1	38.9 ± 9.2 (0.100, 0.095)	3.7 ± 2.5 (0.952, 0.246)
	2	1.5	48.5 ± 4.2 (**<0.001**, **0.013**)	9.5 ± 2.6 (**0.006**, **0.011**)
		1	39.5 ± 6.3 (**0.009**, 0.612)	6.3 ± 3.7 (0.133, 0.115)
2		1	33.3 ± 4.4 (0.155, 0.143)	3.0 ± 1.3 (0.819, 0.842)
Clinical SFRT plans			30.9 ± 3.3	2.5 ± 2.8
Standard lattice plans			35.9 ± 7.0	1.8 ± 0.2

### 3.5 Best‐scoring pattern

Overall, the C = 3 cm, S = 2 cm, and P = 1.5 cm differential lattice pattern outperformed the clinical SFRT plans, and the other differential hole‐size patterns presented here. This lattice pattern resulted in the most significant increases in D_50%,_ V_50%_, and D_mean_ compared to the clinical SFRT plans and standard lattice patterns. Additionally, this pattern has demonstrated a statistical improvement in the ablative doses delivered to the tumor, such as AVP and V_15Gy_. Also, on average, this pattern had the highest recorded dose metrics across all 35 patients compared to all the other SFRT patterns, except for the PVDR metric (Table 1). However, when evaluating nearby OAR doses, this pattern wasn't superior to the other differential hole‐size patterns. Still, this pattern did demonstrate decreases in maximum doses to nearby OARs and decreases in D_2cm_ when compared against the clinically delivered SFRT plans.

## 4 DISCUSSION

This study aimed to demonstrate the feasibility of deploying a differential hole‐size lattice pattern when utilizing SFRT to treat large unresectable tumors, including deep‐seated bulky masses. This differs from how traditional uniform hole size and spacing SFRT is commonly implemented, which consists of having the high‐dose regions planned to be of equal size throughout the tumor volume. Eleven differential hole‐size patterns were investigated and benchmarked against the clinically delivered SFRT plans and the standard uniform lattice pattern.

An interesting result was that tumor dose metrics such as D_50%_, D_mean_, and V_50%_ seem to correlate with the lattice volume, not with the number of lattice spheres packed in the tumor volume. This contradicts conventional thinking with SFRT, in which the goal has commonly been to pack as many lattice vertices within the tumor as possible, as this will increase the high dose volume.^30^ However, due to using a differential hole‐size pattern, more spheres don't equate to more lattice volume, as demonstrated in Figures 3 and 4. Due to the lack of standard SFRT planning practices, with many cancer clinics and institutions using different spacings and vertex diameters from one another, reporting the lattice volume might be more appropriate rather than reporting the number of lattice vertices.^22^, ^31^, ^32^

Further analyzing the tumor dose metrics, most differential lattice patterns showed a dosimetric advantage over the standard lattice pattern across all SFRT treatment sites. However, dosimetrically, only one pattern (C = 3 cm, S = 2 cm, P = 1.5 cm) outperformed all other lattice configurations. It showed a statistically significant increase in tumor dose metrics compared to the clinically delivered SFRT plans. This is also demonstrated in Table 1, as compared to all other differential hole‐size lattice patterns, including the clinical SFRT pattern and standard lattice pattern, the C = 3 cm, S = 2 cm, P = 1.5 cm pattern performed best on average when evaluating the D_5%_, D_10%_, D_50%,_ D_mean_, and D_90%_. It should be noted that this increase in D_10%_ and D_90%_ results in the lowest average PVDR compared to the other differential hole‐size pattern. However, the average PVDR for this lattice pattern was still clinically acceptable at 3.83 ± 2.40. Evaluating how these patterns will perform when analyzing normal tissue dose revealed expected trends. Increasing the spacing or decreasing the peripheral vertex diameter did show a trend of lower D_max_ to nearby critical organs. However, no one pattern outperformed the rest comparatively. Using a 3 cm spacing did result in statistically significant reductions in D_max_; however, a 4 cm spacing demonstrated the smallest D_max_ out of all lattice patterns. Overall, all the differential hole‐size patterns demonstrated a reduction in normal tissue dose compared to the clinically delivered SFRT plans. Still, they did not outperform the standard lattice pattern plans. Surpassing the clinically delivered SFRT plans was expected, as these differential hole‐size patterns were inversely optimized instead of being forward‐planned like the clinical MLC‐based SFRT plans, which allows these novel patterns to better spare nearby OARs if needed. The standard lattice pattern demonstrating lower maximum doses to nearby critical organs was due to the differential hole‐size patterns providing a larger dose to the tumor, as shown in Figures 6, 7, and 8. The consequence of this larger dose being delivered could be more extensive intermediate dose spillage, as shown in Tables 2, 3, and 4 when evaluating D_2cm_, in which the standard lattice pattern for most cases had lower values of D_2cm_.

Moreover, this study investigated the enhanced ablative dose that a differential hole size may provide for SFRT patients. We introduced two metrics to evaluate SFRT treatments: AVP and V_15Gy_. The AVP reveals how much of the high dose (V_50%_) can be attributed to ablative damage (V_75%_), while V_15Gy_ reveals how much the target receives the prescription dose to peak doses. These two new metrics correlate to the lattice volume each lattice pattern deployment can produce. The top two patterns with the largest lattice volume (C = 4 cm, S = 3 cm, P = 2 cm and C = 3 cm, S = 2 cm, P = 1.5 cm) resulted in the largest AVP and V_15Gy_. Compared to the clinical SFRT plans, all the differential hole‐size patterns outperformed the clinical SFRT plans and the standard lattice pattern when evaluating V_15Gy_. This is the benefit of planning for a larger diameter lattice vertex to be placed in the centroid of the tumor. Having more volume receive this high dose area may contribute to an increased therapeutic ratio and better tumor control rate or relief of tumor burden while stimulating an increased bystander effect,^6^, ^33^ potentially increasing the clinical outcomes of SFRT in the future. Increasing the sphere size at the tumor center via this novel differential hole‐size SFRT method can safely deliver a higher biologically effective dose to the tumor core for large and bulky unresectable tumors. The SCART or SBRT‐PATHY technique also follows this line of similarity of delivering a large central ablative dose to the tumor. However, using a differential hole‐size pattern differs from PATHY by still delivering other smaller spheres of high doses toward the periphery of the tumor.^27^, ^28^ However, this method is still in the development phase as an SFRT treatment option. So, this technique needs prospective clinical trials, perhaps a multi‐institutional trial, of using the differential hole‐size SFRT patterns for patients with large and bulky tumors to compare with the clinical outcomes among other SFRT techniques.

These findings suggest that select patients may benefit from these novel differential lattice spacing patterns. However, not all patients can be planned with all the patterns demonstrated in this study. It was observed that highly irregular tumors had difficulty packing more than one lattice vertex with most to all the differential hole‐size patterns. We suggest this technique be deployed for SFRT patients with highly spherical tumors. Additionally, we observed that this technique may not be helpful for tumors with a diameter of less than 6.5 cm, as it was proven difficult to pack more than one lattice vertex within the target. We suggest using the MLC‐fitting‐based planning or standard lattice pattern for tumors less than 6.5 cm in diameter. Tumors with a diameter greater than 6.5 cm could be more easily planned when using 3 cm spacing, while tumors larger than 10 cm in diameter handled being planned much more effectively with a 4 cm spacing. Based on our findings, we highly suggest using the C = 3 cm, S = 2 cm, P = 1.5 cm lattice pattern for select SFRT patients, in which the core of the tumor would benefit from having a larger ablative dose to be delivered.

Another important finding was that these differential lattice patterns demonstrated lower values of D_max_ to nearby critical organs and a sharper dose fall‐off, as shown with D_2cm_ compared to the clinical SFRT plans. It is important to note that further research should investigate the feasibility of escalating the SFRT prescription dose while maintaining normal tissue dose to enhance the therapeutic ratio for select patients. While our study investigated differential hole‐size patterns, ongoing research also explores the possibility of using a differential spacing pattern, which may further reduce the maximum dose to adjacent OARs. As shown in our results, a larger spacing resulted in lower values of D_max_, so having a larger spacing be applied closer to OARs may be beneficial for further sparing the critical organs.

One limitation of this study is that this work was a retrospective simulation study. As of now, no SFRT patients have been treated using this differential hole‐size technique. Although, we aim to utilize this technique to treat our future SFRT patients and perform patient follow‐up study to analyze the effectiveness of these patterns. Additionally, only 30 patients were included in this study's cohort with treatment sites of HN, abdominal/pelvis, and chest/lung. Including patients with a broader range of tumor sites including extremities and a wider range of sizes may provide more information on how to best utilize these differential hole‐size patterns. Another concern of the limited patient cohort is the unknown reproducibility of the dose distributions and metrics across a larger patient population. As seen in Table 1, we observed variability in reproducibility in reported dose metrics for different differential hole‐size patterns, as evidenced by their standard deviations across dose metrics and patterns. Thorough efforts are required to develop and report such parameters in a prospective clinical trial to ensure accordance with recommendations put forward by the RSS SFRT working group.^29^

A standard lattice pattern consisting of a constant vertex diameter throughout the tumor volume may only be a suitable SFRT implementation for certain patients. Our study has explored the potential utility of having nonuniform SFRT lattice patterns and the potential benefits this may bring to patients with large, unresectable tumors, including deep‐seated bulky masses. For select SFRT patients where areas of hypoxic regions or radioresistant tumors are known in multimodality imaging, a large core diameter sphere(s) can be placed in those areas, which may be highly beneficial in improving the tumor local control rate. Additionally, delivering a higher ablative dose to the tumor will enhance the direct cell kill, potentially increasing local control and the potential for indirect cell kill, which may further sensitize the tumor for combination radiation therapy and systemic therapy.

## 5 CONCLUSION

This study investigated the feasibility and potential dosimetric advantage of utilizing a differential hole‐size pattern when treating SFRT patients with large and bulky tumors including deep‐seated tumor masses. This study has found that using a differential hole‐size pattern generally results in a larger volume receiving an ablative dose when compared against clinical SFRT plans and the standard lattice pattern. The differential hole‐size patterns also demonstrated a reduced dose to nearby OARs compared to the clinical SFRT plans. Although these patterns gave similar results to the clinical SFRT plans when evaluating PVDR, D_50%_, D_mean_, and V_50%_ on average, these novel differential hole‐size patterns outperformed the standard lattice pattern. Overall, these new patterns produced clinically acceptable SFRT plans that enhance the ablative dose delivered to the tumor while maintaining normal tissue doses. These SFRT patterns have allowed for an enhanced dose to the tumor's core while maintaining the dose to nearby OARs, potentially improving the therapeutic ratio for SFRT treatments. For select SFRT patients, we plan to implement the differential lattice patterns in our clinic.

## AUTHOR CONTRIBUTIONS

Damodar Pokhrel, PhD, designed the 3D MLC‐based SFRT treatment method, developed MLC fitting algorithm and generated clinical SFRT plans. For assessing the dosimetric impact, Damodar Pokhrel, PhD and Josh Misa, MS conceptulaized, generated simulated differential hole‐size SFRT plans, collected and analyzed the data. William St. Clair, MD, PhD provided radiation oncology clinical expertise. Josh Misa, MS and Damodar Pokhrel, PhD drafted the prelimianary manuscript. All co‐authors revised, edited, and approved the final document for submission.

## CONFLICT OF INTEREST STATEMENT

The authors declare no conflicts of interest.

## Data Availability

No data available on request due to privacy/ethical restrictions.
